# Value of ^18^F-FDG PET/CT in patients with hepatic metastatic carcinoma of unknown primary

**DOI:** 10.1097/MD.0000000000023210

**Published:** 2020-12-11

**Authors:** Yuekai Li, Fengcai Li, Xin Li, Lili Qu, Jiankui Han

**Affiliations:** a Department of Nuclear Medicine, Cheeloo College of Medicine; b Department of Hepatology, Qilu Hospital of Shandong University, Jinan, China.

**Keywords:** fluorodeoxyglucose, hepatic metastatic carcinoma of unknown primary, positron emission tomography/computerized tomography

## Abstract

**Purpose:**

This retrospective study aimed to investigate the clinical value of -deoxy-2-(^18^F)-fluorodeoxyglucose positron emission tomography/computed tomography (^18^F-FDG PET/CT) in detecting primary lesions of hepatic metastases.

**Methods:**

A total of 124 patients with hepatic metastatic carcinoma of unknown primary underwent whole body ^18^F-FDG PET/CT imaging. According to the final diagnoses for both primary sites and hepatic metastases that were confirmed either histopathologically or by clinical follow up, all patients were divided into 4 groups: a true positive group (TP, 95 cases), a false positive group (FP, 9), a true negative group (TN, 8) and a false negative group (FN, 12).

**Results:**

The TP rate of primary lesions, detected by ^18^F-FDG PET/CT, was 76.61%, the FP rate 7.26%, the TN rate 6.45% and the FN rate 9.68%. The sensitivity, specificity, positive predictive value, negative predictive value and accuracy of ^18^F-FDG PET/CT in the detection of primary tumors were 88.78%, 52.94%, 91.35%, 40%, and 83.06%, respectively. Accurate diagnosis groups (TP, TN) showed a significantly higher SUVmax (standard uptake maximum value) level than that in error diagnosis groups (FP, FN). The SUVmax between hepatic metastases and primary lesions had a positive correlation. The primary tumor sites of hepatic metastases were mainly located in the gastrointestinal organs and the lungs.

**Conclusions:**

Whole body ^18^F-FDG PET/CT imaging was sensitive for detecting primary sites/lesions with hepatic metastatases of unknown primary, especially when the SUVmax of hepatic metastases were greater than 4.7.

## 1 Introduction

Carcinoma of unknown primary (CUP) refers to the presence of a metastatic disease that was documented in the absence of an identifiable primary lesion. The criteria for CUP diagnosis includes a biopsy proved metastatic malignancy, absence of a primary tumor after a thorough examination and normal laboratory tests, including complete blood count and chemistry, serum tumor markers, chest, abdomen, and pelvis computer tomography (CT) scans, and ultrasound.^[1,2]^ CUP is observed in approximately 2.3% to 4.2% of newly diagnosed cancer patients.^[3]^ Hepatic metastatic carcinoma of unknown primary (HCUP) refers to a CUP that occurs in the liver. Because of the liver's rich blood supply, hepatic metastases can be derived from a variety of tissues and organs, such as the digestive tract, lungs, kidneys, thyroid, and mammary gland, or the musculoskeletal system. The early diagnosis and staging of the tumor primary lesions are particularly important for patient prognosis.^[4]^

Several studies have indicated that 2-deoxy-2-(^18^F)-fluorodeoxyglucose positron emission tomography/computed tomography (^18^F-FDG PET/CT) is a valuable diagnostic tool in the detection of the primary lesions in patients with CUP syndrome.^[5,6]^ However, there were relatively few studies on the role of PET/CT in detecting the primary lesions of hepatic metastases.^[7]^^18^F-FDG PET/CT is the most advanced clinical application of molecular imaging technology. It can provide whole body imaging on both morphological and functional aspects. It can greatly improve the detection rate of lesions by providing a convenient qualitative diagnosis of the pathological changes, and a dynamic observation of the effect of the clinical treatment.^[8]^

This retrospective study aimed at investigating the clinical value of ^18^F-FDG PET/CT in detecting HCUP primary lesions.

## 2 Materials and methods

This was a single-institution study approved by the Ethical Committee. Written informed consents were obtained from all patients.

### 2.1 Patients

A total of 552 patients with a highly suspicious diagnosis of HCUP underwent whole body ^18^F-FDG PET/CT imaging in our PET/CT center from March 2010 to September 2018. However, only 124 eligible patients were eventually enrolled into the study. The final diagnoses for primary sites and hepatic metastases were confirmed either histopathologically or by clinical follow up which included other imaging methods. None of the patients had a history of cancer, received chemotherapy and/or radiation therapy prior to the FDG-PET/CT examination. The patients included 76 male cases and 48 female cases, whose age ranged from 25 to 82 years, with an average age of 56 years. In these cases, the hepatic metastases were pathologically classified into 69 cases of adenocarcinoma, 20 cases of squamous carcinoma, 6 cases of small cell carcinoma, 4 cases of neuroendocrine carcinoma, and 25 cases of other types.

### 2.2 ^18^F-FDG PET/CT imaging

All patients underwent ^18^F-FDG PET/CT imaging after more than 6 hours of fasting. Before ^18^F-FDG injection, the patients’ blood sugar levels were less than 8.3 mmol/L. After intravenous administration of ^18^F-FDG 3.7 MBq/kg, the patients rested in a quiet room for 45 to 60 minutes before imaging.

The imaging instrument model was a GE Discovery STE16 (GE Healthcare) and ^18^F- FDG was produced by a PET/CT center. The cyclotron's model was a GE Minitrace. The image acquisition ranged from parietal to femoral and when necessary, imaging of lower limbs was also performed. We used 16 row helical CTs through scanning with the following conditions: tube voltage (body 120 kv, craniocerebral 160 kv), tube current (body 110 mA and craniocerebral 260 mA), 3.75 mm thick; PET collection every bed time was 3 minutes, the whole body scanning needed 6 to 7 beds. Using the viewpoint method, the scan data were rebuilt into an image fusion, resulting in transaxial, coronal, and sagittal CT, PET, and PET/CT image fusion.

### 2.3 Image analysis

All PET/CT images were independently reviewed by at least 2 experienced nuclear medicine physicians. The PET/CT image analysis was divided into 2 methods:

(1) A visual inspection method: first, we evaluated the CT, PET, and PET/CT images to exclude interference factors, such as the existence of metal artifacts and respiratory mobility. Second, we analyzed the characteristics of the PET/CT images (such as location, morphology, number, density, and FDG uptake level) and then selected typical images for preservation.
(2) A quantitative analysis: we drew interested areas of both inside and outside liver lesions, obtained a standard uptake maximum value (SUVmax) and analyzed the interactive relationships among different pathological types and different primary sites.

The analysis of hepatic metastases and primary lesions were based on the images’ qualitative visual interpretation. The criterion for hepatic metastases was based on the FDG hyper metabolism at the site of pathological changes on the CT^[9,10]^ or on the marked focal hyper metabolism at physiological uptake sites and despite the absence of signs of pathology on the CT. The identification of the primary site was based on the presence of a focal abnormal tracer uptake in an organ. The identification procedure was more difficult in cases of disease with many foci in different organs. The distribution of the pathological lesions, a prior knowledge of the pattern of spread of different tumors and the patient's history, were taken into consideration. The fused FDG-PET/CT images were analyzed in at least 3 planes (coronary, sagittal, and axial) – of the gray scale color table for PET.^[6,11–13]^

### 2.4 Statistical analysis

The statistical analysis was performed using the IBM SPSS Statistics 21 software and GraphPad Prism 5. The gold standard was based on the pathological verification of the sites (both primary and liver metastatic) suggested by FDG PET/CT. When the pathological verification could not be performed, results of further procedures or clinical follow ups (at least 1 year or until death) were accepted.

All patients were divided into 4 groups. In the true positive (TP) group, the pathological results of primary lesions, suggested by PET/CT, were consistent with those of hepatic metastases. In the false positive group (FP), the pathological results of primary lesions, suggested by PET/CT, were inconsistent with those of hepatic metastases. In the true negative (TN) group, the PET/CT did not indicate a clear primary site; however, after at least 1 year of follow-up or until patient's death, no specific primary lesions were observed. In the false negative (FN) group, PET/CT did not indicate a clear primary site, but the exact primary lesions were found by further procedures or clinical follow ups (at least 1 year or until patient's death). Sensitivity, specificity, positive, and negative predictive values and accuracy were calculated using the following standard statistical formulas:

Sensitivity = TP/(TP + FN), Specificity = TN/(TN + FP), Positive predictive value = TP/(TP + FP), Negative predictive value = TN/(TN + FN), Accuracy = (TP + TN)/(TP + FP + TN + FN).

The Chi-squared test (χ^2^) was used to compare the differences between any 2 groups. The Spearman rank correlation coefficient was used to evaluate the correlation between primary lesions SUVmax and hepatic metastases SUVmax. Logistic regression was used to determine the potential factors that affect the accuracy of primary lesions detection in hepatic metastases by PET/CT of HCUP patients. Covariates with *P* < .05 were incorporated into the multivariate Logistic regression analyses to determine the independent risk factors that affect the accuracy of PET/CT in the diagnosis HCUP patients. The Receiver operating characteristic curve was used to evaluate the diagnostic value of hepatic metastases SUVmax for accuracy of detecting primary lesions in hepatic metastases by PET/CT in HCUP patients. A *P*-value of less than .05 was considered statistically significant.

## 3 Results

### 3.1 General characteristics of the study's population

According to patient groups (TP, FP, TN, FN), there were 95 cases in TP, 9 cases in FP, 8 cases in TN and 12 cases in FN. ^18^F-FDG PET/CT sensitivity, specificity, positive predictive value, negative predictive value, and accuracy in the detection of primary tumors were identified as 88.78%, 52.94%, 91.35%, 40%, and 83.06%, respectively.

Among the pathological types of hepatic metastases, the percentage of metastatic adenocarcinomas in the TP group was the highest (60.00%) followed by metastatic squamous carcinomas (17.89%). In the other 3 groups, metastatic adenocarcinoma was also the most common pathological type. In the TN group that included 8 cases, that were confirmed by follow-up and pathology, most of the cases were metastatic adenocarcinomas and nodular hepatic carcinomas (Table 1).

**Table 1:** The pathological types of hepatic metastases and its distribution in each group.

Patient groups	Pathological types of hepatic metastatases	Number of cases	Constituent ratio in each group (%)
TP, 95, (76.61%)	Metastatic adenocarcinoma	57	60.00
	Metastatic squamous cell carcinoma	17	17.89
	Small cell carcinoma	6	6.32
	Neuroendocrine carcinoma	4	4.21
	Malignant melanoma	3	3.12
	Medullary carcinoma	2	2.10
	Rhabdomyosarcoma	1	1.05
	Sarcomatoid carcinoma	1	1.05
	Mesenchymoma	1	1.05
	Pleomorphic lobular carcinoma	1	1.05
	Thecoma	1	1.05
	Malignant peripheral schwannoma	1	1.05
FP, 9, (7.26%)	Metastatic adenocarcinoma	3	33.33
	Nodular hepatic carcinoma	2	22.22
	Cholangiocarcinoma	2	22.22
	Lymphoma	2	22.22
TN, 8, (6.45%)	Metastatic adenocarcinoma	4	50.00
	Nodular hepatic carcinoma	3	37.25
	Lymphoma	1	12.50
FN, 12, (9.68%)	Metastatic adenocarcinoma	5	41.67
	Clear cell carcinoma	2	16.67
	Signet ring cell cancer	2	16.67
	Malignant mesothelioma	1	8.33
	Rhabdomyosarcoma	1	8.33
	Transitional cell carcinoma	1	8.33

FN = false negative, FP = false positive, TN = true negative, TP = true positive.

In the TP group that included 95 cases, the primary sites were mainly in the gastrointestinal tracts, lungs and the female reproductive system. In the FP group that included 9 cases, the primary focal areas diagnosed by ^18^F-FDG PET/CT were mostly located in the lungs. In the FN group that included 12 cases, that were confirmed by followed ups and pathology reports, the original sites were located in the kidneys (2 cases), pancreas (2 cases), stomach (2 cases, signet ring cell carcinoma), lung (1 case), ureter (1 case), prostate (1 case), peritoneum (1 case), rectum (1 case) and the right vastus intermedius (1 case) (Table 2).

**Table 2:** Primary sites of hepatic metastases and its distribution in each group.

Patient groups	Site of primary lesions	Number of cases	Constituent ratio in each group (%)
TP, 95, (76.61%)	Stomach	16	16.84
	Lung	13	13.68
	Colon	15	15.79
	Small intestine	3	3.16
	Female reproductive system	10	10.53
	Rectum	8	8.42
	Pancreas	3	3.16
	Breast	5	5.26
	Esophagus	10	10.52
	Laryngopharynx	2	2.10
	Pleura	2	2.10
	Other	6	6.32
FP, 9, (7.26%)	Lung	3	33.33
	Colon	2	22.22
	Stomach	1	11.11
	Esophagus	1	11.11
	Small intestine	1	11.11
	Common bile duct	1	11.11
TN, 8, (6.45%)	None		
FN, 12, (9.68%)	Stomach	2	16.67
	Kidney	2	16.67
	Pancreas	2	16.67
	Rectum	1	8.33
	Ureter	1	8.33
	Lung	1	8.33
	Peritoneum	1	8.33
	Vastus intermedius	1	8.33
	Prostate	1	8.33

FN = false negative, FP = false positive, TN = true negative, TP = true positive.

### 3.2 SUVmax value analysis for different groups of HCUP

**Figure 1:**
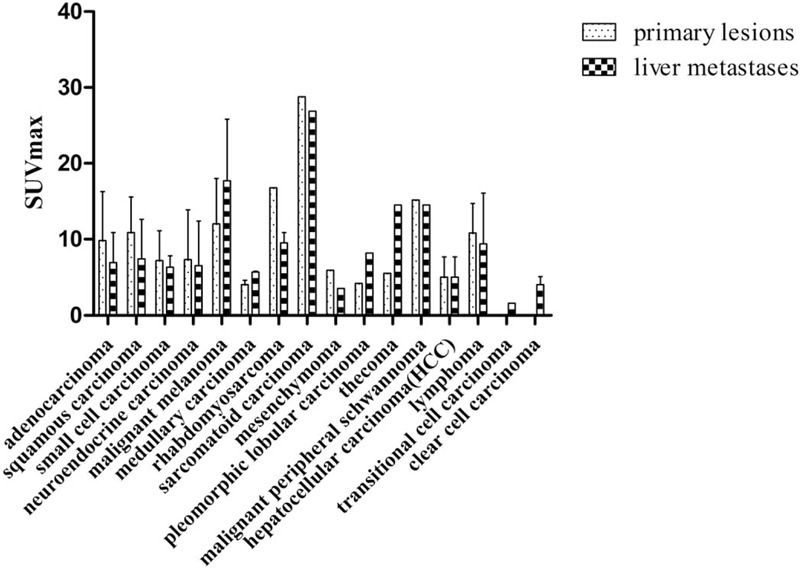
The standard uptake maximum value of different pathological types.

**Figure 2:**
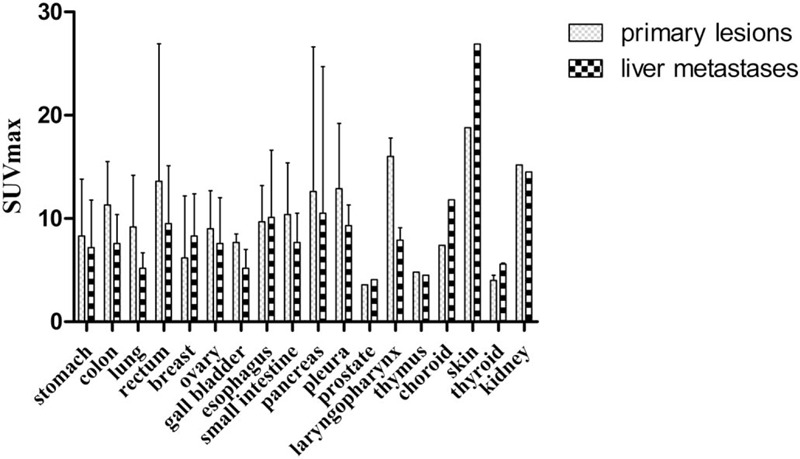
Standard uptake maximum value of different primary sites.

**Figure 3:**
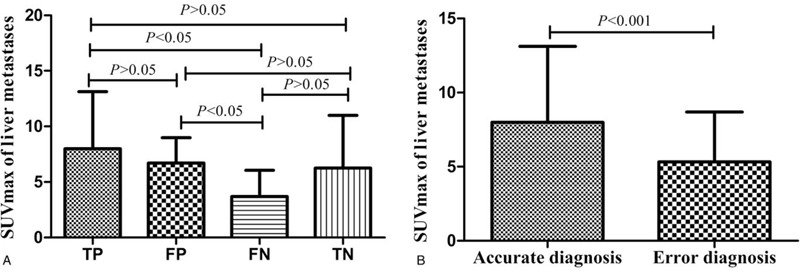
Standard uptake maximum value comparison of 4 groups (A), standard uptake maximum value comparison between the accurate diagnosis group and error diagnosis group (B).

We compared the SUVmax of hepatic metastases in patients with different pathological types and found no statistical differences (*P* = .100) (Fig. 1). In addition, we compared the SUVmax of different primary sites and found no statistical differences either (*P* = .310) (Fig. 2). By analyzing the SUVmax value, there were statistical differences between the TP and FN groups, and the FP and FN groups. However, there were no statistical differences between the TP and FP groups, the TP and TN groups, the FP and TN groups, and the TN and FN groups (Fig. 3A). We combined the TP and TN groups as the accurate diagnosis group, the FP and FN groups as the error diagnosis group, and compared the SUVmax of hepatic metastases in the 2 groups, and found that the accurate diagnosis group had a significantly higher SUVmax level [6.0 (4.1; 9.8)] than that in the error diagnosis group [4.3 (2.9; 6.9), *P* < .001] (Fig. 3B).

### 3.3 SUVmax correlation analysis of primary lesions and hepatic metastases

**Figure 4:**
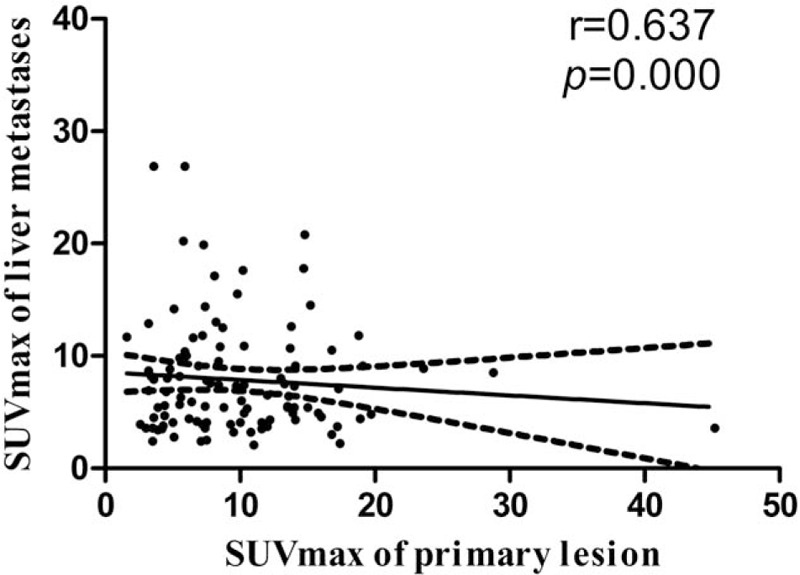
Standard uptake maximum value correlation analysis of primary lesions and hepatic metastases.

We compared the correlation between SUVmax of hepatic metastases and SUVmax of primary lesions and found a positive correlation between the SUVmax of hepatic metastases and the SUVmax of primary lesions (*r* = 0.637, P = .000) (Fig. 4).

### 3.4 The SUVmax of hepatic metastases is an independent risk factor for the efficacy of PET/CT in finding primary lesions.

We used univariate and multivariate logistic analysis to identify the potential factors affecting the accuracy of detecting primary lesions in hepatic metastases by PET/CT (Table 3). Univariate logistic analysis showed that the SUVmax of hepatic metastases [odds ratio (OR) = 1.243, *P* < .05) and the pathological type of hepatic metastases (OR = 0.858*, P* < .05) significantly correlated with PET/CT diagnostic accuracy. We included these 2 factors into multivatiate logistics regression and confirmed that both SUVmax of hepatic metastases (OR = 1.244, *P* < .05) and pathological type of hepatic metastases(OR = 0.848, *P* < .05) were independent risk factors affecting the diagnostic accuracy of PET/CT. The SUVmax of hepatic metastases was more important in predicting the diagnostic efficacy of PET/CT due to the invasive nature of pathology and its absence before PET/CT.

**Table 3:** Uni- and multivariate logistic analysis of factors affecting the accuracy of detecting primary lesions by 2-deoxy-2-(^18^F)-fluorodeoxyglucose positron emission tomography/computed tomography in patients with hepatic metastatic carcinoma of unknown primary.

	Univariate			Mutlivariate		
Variable	OR	95%CI	*P*	OR	95%Cl	*P*
Male(%)	1.323	0.492–3.557	.580			
Age(yr)	0.987	0.945–1.032	.573			
Primary lesion	1.077	0.885–1.312	.458			
SUVmax of primary lesion	0.964	0.880–1.056	.428			
SUVmax of hepatic metastases	1.243	1.039–1.488	.017	1.244	1.041–1.485	.016
Pathological type	0.858	0.777–0.946	.002	.848	0.762–0.944	.003

CI = confidence interval, ^18^F-FDG PET/CT = 2-deoxy-2-(^18^F)-fluorodeoxyglucose positron emission tomography/computed tomography, HCUP = hepatic metastatic carcinoma of unknown primary, OR = odds ratio, SUVmax = standard uptake maximum value.

### 3.5 The value of SUVmax of hepatic metastases for the accuracy of primary lesions detection in hepatic metastases by PET/CT

**Figure 5:**
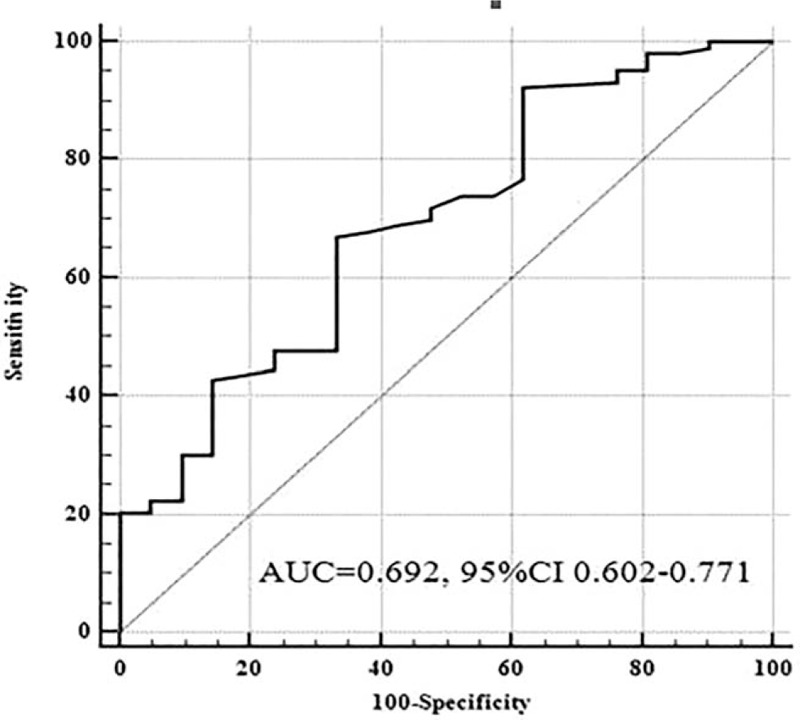
Hepatic metastases standard uptake maximum value for predicting the efficacy of Positron emission tomography/computed tomography in finding primary lesions.

The accuracy of PET/CT in detecting primary lesions of hepatic metastases was 83.06% (103/124). The accurate diagnosis group showed a significantly higher SUVmax level [6.0 (4.1; 9.8)] with the error diagnosis group [4.3 (2.9; 6.9), *P* < .001] (Fig. 3B). The AUROC for hepatic metastases SUVmax was 0.692 (SE 0.0638, 95% confidence interval 0.602–0.771) (Fig. 5). The 4.7 cut-off point for hepatic metastases SUVmax with a sensitivity of 66.99% and a specificity of 66.67% was selected to discriminate the efficacy of PET/CT in finding primary lesions. Furthermore, the accuracies of PET/CT in finding primary lesions in HCUP patients with the level of SUVmax > 4.7 and ≤ 4.7, were 90.8% (69/76) and 70.8% (34/48) (*P* < .05). In summary, hepatic metastases SUVmax might indicate the efficacy of PET/CT in finding primary lesions.

## 4 Discussion

As a dual imaging system based on the morphology and function of the body, PET/CT can be used for a more comprehensive assessment of the patient, that could provide important clinical significance in the diagnosis of tumor staging and treatment monitoring.^[14,15]^ Compared with traditional means of imaging (such as CT, magnetic resonance, and ultrasound), it is easier to find, at an early, irregular parts, and primary tumor lesions using PET/CT. More and more clinical doctors have realized the clinical value of PET-CT in determining the original site of a metastatic tumor.^[16,17]^ Although some scholars have previously reported on this, most of the studies did not investigate PET-CT specific application for HCUP.^[25,26]^

In this study, the TP rate in HCUP patients was 76.61%, the number of pathological type for adenocarcinoma highly ranked and most of the primary sites were gastrointestinal tracts (56.86%), which was consistent with previous reports in the literature.^[18]^ Although there are several clinical routine inspection methods for the digestive tract, such as endoscopic ultrasound, CT and magnetic resonance, there are still some primary lesions that could not be found. This limitation may be due to the following points:

(1) the change of primary lesion forms is not obvious or not typical, and therefore, it is difficult to find those under endoscopy macroscopic field of vision.

**Figure 6:**
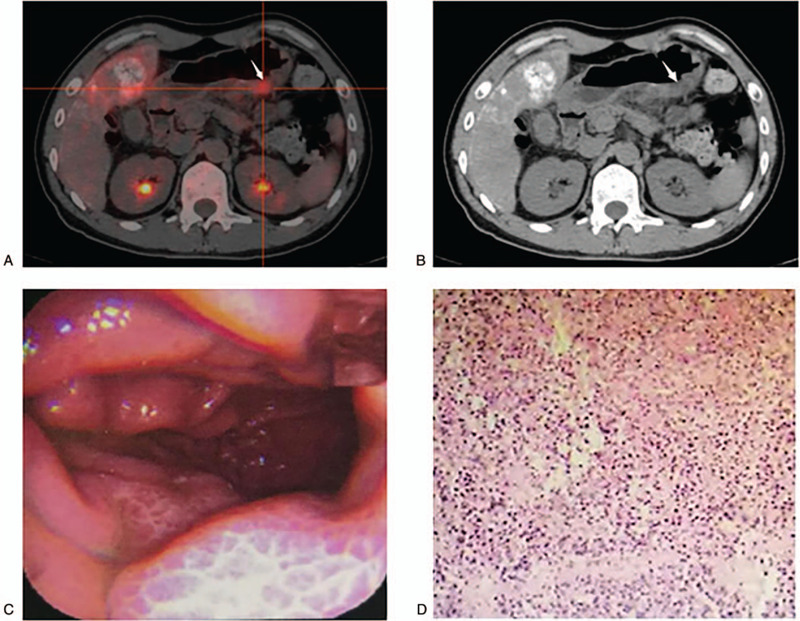
(A) Positron emission tomography/computed tomography fusion image showed a focal abnormal uptake of 2-deoxy-2-(^18^F)-fluorodeoxyglucose in the greater curvature side which was indicated by the white arrow. (B) Focal gastric wall was a bit thicker, and its density was slightly lower on the plain computed tomography image indicated by the white arrow. (C) Gastroscope only showed the local rough slight uplift, it was not easy to distinguish lesion gastric mucosal tissue with naked eyes. (D) Final pathological results suggested neuroendocrine tumor, G3.

As shown in Figure 6, the middle-aged male patient's first symptoms were nausea, vomiting and upper abdominal discomfort, and for the first time the gastroscope only showed chronic gastritis. However, the abdominal CT showed that the liver had multiple metastases and lymph nodes enlargement in the upper abdominal part. The tumor markers carcinoembryonic antigen, alpha fetoprotein, and carbohydrate antigen 199 stayed in normal ranges; carbohydrate antigen 125 was 19.76U/mL (normal for 0–5U/mL). Therefore, an additional diagnosis was required using PET/CT examination and which showed that some parts of the gastric wall was a bit thicker than normal and ranging at approximatively 2.1 × 1.8 cm. Its density was slightly lower than the normal parts and its maximum standardized uptake value (SUVmax) was 4.6. In the PET/CT report, we highly suspected primary lesions, located in the gastric body side along the greater curvature, and advised another gastroscopy examination. On the secondary gastroscopy, in the PET/CT shown lesion site, we punctured 6 biopsy specimens that were sent for pathologic examinations. However, the lesions in the perspective of a gastroscope only showed a local rough slight uplift and it was difficult to distinguish the gastric mucosal tissue lesion with naked eyes. The final pathologic results showed the presence of a neuroendocrine tumor, G3. The parts of the primary lesions in patients with distant organ and self-conscious symptoms were not obvious and the clinician had difficulties diagnosing them as conventional examinations may easily miss the primary lesion. For instance, in the study, there were 2 cases with primary lesions located in hypopharynx, 2 cases in the thyroid, 2 cases in the pleura, 1 case in the bladder, 1 case in the choroid and 1 case in the thymus. Because PET/CT examination was based checking the whole-body, its scope was comprehensive and detailed with an increased detection rate of the primary tumor lesion, consistent with previous reports.^[19]^ Furthermore, the parts of the primary lesion locations were relatively hidden, and the density change was not obvious, and therefore, traditional imaging examinations had difficulties distinguish the lesions from normal tissue structures. In this study, there were 3 cases of primary lesions located in the start of the ministry of the ascending colon, 3 cases in the small intestine, and 3 cases in the pancreas. If the change in the lesions form of the start of the ascending colon was not obvious, it was difficult to effectively identify it with the intestinal plica and the bending of normal physiological changes.^[20]^ Moreover, due to the variable morphology of the small intestinal and lack of effective means of endoscopy in some institutions, malignant lesions in the small intestine could possibly be missed.^[21]^ On the other hand, most of the intestinal malignant lesions were characterized by FDG high metabolic activity,^[22,23]^ which was more striking and intuitive, and easier in locating the nature of the lesion.

In this study, the constituent ratio of the FP group was 7.26% and lung primary malignant lesions misdiagnosis constituted the majority with 33.33%. Among these cases, 2 cases were pulmonary tuberculosis with metastatic liver cancer and 2 cases were lung compliance pseudotumors with liver metastasis. In the ^18^F-FDG PET–CT imaging, many kinds of lesions, such as primary lung cancer, tuberculosis and inflammatory pseudotumor could absorb the imaging agent ^18^F-FDG, with tuberculosis stove and inflammatory pseudotumor inflammatory lesions often having the highest intake. While most hepatic metastases highly intake ^18^F-FDG, their metabolic activity was identical, which was likely to mislead doctors’ diagnosis in thinking that there was a causal relationship between the 2. In these circumstances, subtle CT features and pathological changes of delayed imaging SUV values change, become particularly important. Patel VK et al^[24]^ suggested that for atypical pulmonary space-occupying lesions a thin layer scanning and 3D reconstruction and PET delayed imaging, based on conventional PET/CT imaging, should be added to improve the diagnostic accuracy. This is the aspect where further research was needed. In the FN group, the missed diagnosis of kidney primary malignant lesions accounted for 16.67% and the reason may mainly be explained by the following 2 aspects:

(1) The ^18^F-FDG mainly excretes through the kidneys and a large number of radioactive concentration in the urine may be characterized as a diffuse enhancement in the image, which could cover the primary lesion.
(2) Under the circumstances of small lesions of kidney disease and a slight change of shapes, it was difficult to find lesions using only a CT scan to observe density change. To avoid missed diagnosis, referring to the patient existing test results, such as enhanced CT images and abdomen color to exceed), becomes particularly important.

**Figure 7:**
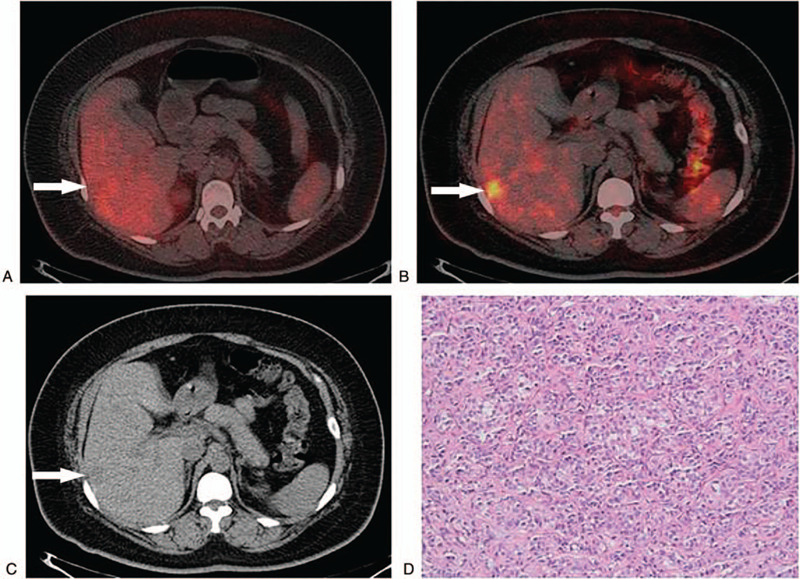
(A). Conventional positron emission tomography/computed tomography fusion imaging showed that the fluorodeoxyglucose uptake level of the lesion in the right lobe of the liver (indicated by the white arrow) was similar to that of normal liver tissue, standard uptake maximum value was 4.5. (B) Two-hour delayed positron emission tomography/computed tomography fusion imaging showed that the fluorodeoxyglucose uptake level of the lesion in the right lobe of the liver (indicated by the white arrow) was significantly higher than that in normal liver tissue, standard uptake maximum value was 7.5. (C) Plain computed tomography imaging showed that the lesion in the right lobe of the liver (indicated by the white arrow) was a quasi-circular and low-density lesion with unclear boundary, the diameter was about 2.6 cm. (D) Final pathological results suggested metastatic adenocarcinoma.

In this study, 22 patients (17.8%) received dual-point PET/CT imaging of the liver. We found that the dual-point PET/CT imaging was meaningful for the detection of intrahepatic lesions. As shown in Figure 7, there was metastatic adenocarcinoma in right lobe of liver. Conventional PET/CT fusion imaging showed that the FDG uptake level was similar to that of normal liver tissue, SUVmax was 4.5. But 2-hour delayed PET/CT fusion imaging showed that the FDG uptake level of the lesion in the right lobe of the liver was significantly higher than that in normal liver tissue, SUVmax was 7.5.

In conclusion, a whole body ^18^F-FDG PET/CT imaging is sensitive for detecting primary sites/lesions with hepatic metastases of unknown primary, especially when SUVmax of hepatic metastases were greater than 4.7.

## Author contributions

**Conceptualization:** Yuekai Li, Jiankui Han.

**Data curation:** Yuekai Li.

**Formal analysis:** Yuekai Li, Fengcai Li, Jiankui Han.

**Investigation:** Yuekai Li, Lili Qu.

**Methodology:** Yuekai Li, Fengcai Li, Xin Li, Jiankui Han.

**Project administration:** Xin Li.

**Resources:** Yuekai Li, Lili Qu.

**Software:** Yuekai Li, Fengcai Li.

**Supervision:** Fengcai Li, Xin Li, Jiankui Han.

**Validation:** Yuekai Li.

**Visualization:** Yuekai Li, Jiankui Han.

**Writing – original draft:** Yuekai Li.

**Writing – review & editing:** Jiankui Han.
